# Development and Validation of a Psychoeducational Video on Depression

**DOI:** 10.7759/cureus.59347

**Published:** 2024-04-30

**Authors:** Mohd Zahiruddin Mohd Khairi, Abqariyah Yahya, Nik Daliana Nik Farid, Rafidah Aga Mohd Jaladin, Nur Amani Natasha Ahmad Tajuddin

**Affiliations:** 1 Department of Social and Preventive Medicine, Universiti Malaya, Kuala Lumpur, MYS; 2 Department of Educational Psychology and Counseling, Universiti Malaya, Kuala Lumpur, MYS; 3 Department of Primary Care Medicine, Universiti Malaya, Kuala Lumpur, MYS

**Keywords:** university students, literacy, depression, video, psychoeducation, validation, development

## Abstract

Background

Depression is an important public health issue that affects many university students worldwide. It causes a wide range of physiological impairments, resulting in high mortality and morbidity rates. Regardless of the severity of the situation, the lack of knowledge about depression prevents them from seeking help. Furthermore, many individuals mistakenly believe that supernatural powers cause mental illnesses, which leads them to seek treatment from traditional healers instead of medical professionals. Therefore, video is a suitable instrument for disseminating information about depression because it corresponds to the younger generations’ preferred method of accessing information. However, there is a scarcity of research on the development of psychoeducational videos aimed at improving depression literacy among university students in Malaysia. Hence, this study was conducted to address this research gap.

Methods

The study was conducted from October 2022 to October 2023, following the guidelines of video production and the principles of psychoeducation. During the preproduction phase, the script was written based on medical reference books and medically reviewed websites. The script’s consistency, relevance, representativeness, and clarity were validated by five subject-matter experts. Subsequently, it was translated into Malay, which is the national language of Malaysia, to create subtitles. This was followed by script breakdown, storyboard and shot list creation, location scouting, blocking, shooting schedule, preparing call sheets, and recruiting talents. The production phase included the activities of voiceover recordings and filming, followed by the post-production phase, which consisted of video editing. Nine subject matter experts and 30 end-users validated the video by assessing its functionality, usability, efficiency, audio-visual style, and setting.

Results

The video script scored an overall item-level content validity index (I-CVI) and scale-level content validity index based on the average method (S-CVI/Ave) value of 1 for the domains of consistency, relevancy, representativeness, and clarity. On the other hand, the video's I-CVI and S-CVI/Ave scores indicated that it met the minimum acceptable value of 0.78 (with a range of 0.78 to 1) and 0.9 (with a range of 0.92 to 0.98) in the domains of functionality, usability, efficacy, audiovisual technique, and setting. Similarly, the video's item-level face validity index (I-FVI) achieved a minimal value of 0.80 (with a range of 0.87 to 1) across all domains following validation by 30 end-users. The video's scale-level face validity index based on the average method (S-FVI/Ave) scored 0.91, 0.96, and 0.97 for functionality, usability, and consistency domains, while the audiovisual technique and setting domains scored 0.94 and 0.98, respectively. The feedback from participants included reducing the video speed to increase subtitle readability, replacing gloomy background music with a more vibrant composition and tempo, and substituting stock videos with footage depicting Asian settings.

Conclusion

The psychoeducational video, titled “Educating University Students About Depression: A Short Documentary,” serves as a valid instrument for disseminating information pertaining to depression. The practical application of this video has the potential to improve depression literacy and mitigate the adverse consequences of depression among university students.

## Introduction

Depression is a mental health disorder marked by severe impairments in mood that affect a person’s emotions, thinking, and ability to perform daily activities [1]. It is one of the leading causes of disability, affecting 322 million individuals worldwide, with the majority residing in Southeast Asia and the Western Pacific region [2]. Research has indicated that university students are more susceptible to developing mental health disorders and are at a greater risk of suffering from depression, anxiety, and stress compared to the general population [3]. According to a study by Wong SS et al. (2023), approximately 53.9% of university students in Malaysia suffer from moderate to severe depression [4]. The study revealed that the frequency of exercise and an individual's personal history of depression were strong indicators of depression [4].

Individuals afflicted with depression are more susceptible to participating in substance and alcohol abuse, as well as smoking. Furthermore, it has been associated with a greater risk of developing cardiovascular diseases, diabetes, and cancer, which are linked to higher mortality and morbidity rates [5]. Additionally, there is a strong association between depression and suicidal ideation, as those struggling with depression are 11 times more likely to commit suicide [6]. Despite the severity of the situation, Malaysian university students demonstrated a poor help-seeking attitude, with an estimated 10% to 25% of eligible students failing to utilize mental health services [7]. In addition, people hold numerous myths and misconceptions about mental illness, ascribing its causes to spirits and supernatural occurrences [8]. As a result, many individuals depend on the services provided by traditional healers instead of seeking medical assistance from trained professionals [8,9].

In recent years, there has been a rise in the utilization of digital video interventions to address the issue of depression [10]. This platform is well-suited for disseminating information about depression to young adults, as it aligns with their preferred mode of accessing information [11]. The reason for this situation is connected to the evolution of the social media landscape. Research indicates that the majority of young adults primarily use YouTube as their main social media site, followed by TikTok. This trend can be attributed to the recent rapid increase in the popularity of short videos [12].

Therefore, digital video interventions can serve as an effective tool for conducting mental health interventions. The use of digital video can improve students’ engagement, focus, and understanding of the subject matter. In addition, it enhances students' learning experiences by allowing them to watch the content repeatedly, creating a more in-depth connection to real-life situations through visual representation, and improving memory recall compared to reading [13-15]. This is because the human brain engages in deeper cognitive processing when information is presented via different sensory modalities [16].

While there is an abundance of material about depression available on the internet, only a limited number of these resources have been validated by subject-matter experts. This could pose a threat due to the use of inaccurate information [17,18]. Therefore, intervention programs that use audiovisual methods need to comply with a structured development process that is based on established principles. This is crucial to ensure the accurate dissemination of scientific knowledge and to make it visually engaging. Moreover, this method enables the audience to gain new knowledge and rectify any misconceptions they might have, while simultaneously reducing the risks of the audience disregarding the video [19]. However, to the best of the researcher’s knowledge, there have been limited studies conducted to develop and validate psychoeducational videos with the objective of improving depression literacy among university students in Malaysia. Hence, this study was conducted with the objective of developing and validating a psychoeducational video aimed at improving depression literacy among university students in Malaysia, considering the importance of investigating this topic.

## Materials and methods

Ethical clearance

This study obtained ethical clearance from the Malaysian Research Ethics Committee (MREC) of the Ministry of Health Malaysia, (NMRR ID-22-01198-7C2) and the University of Malaya Research Ethics Committee (UMREC), with reference number UM.TNC2/UMREC_2427. It was conducted in compliance with the established ethical standards and regulations set by the UMREC. Participation in this research was completely voluntary, and only those who provided written consent were allowed to participate. Each participant had the right to withdraw from the study at any given moment, as well as to retract any personally identifiable data.

Study setting and population

This study was conducted at Universiti Malaya, a public university in Kuala Lumpur, Malaysia, from October 2022 to October 2023. The participants consisted of subject matter experts, including two senior counselors, two public health medicine specialists, two psychiatrists, an educational psychology and counseling professor, and two primary care medicine specialists. The study also included undergraduate students who served as end-users.

Inclusion and exclusion criteria

Subject-matter experts required a minimum of 10 years of professional experience in mental health to be eligible for this study. Experts who did not meet the inclusion criteria, declined to participate, or were unable to participate due to work commitments, sabbatical leave, or other reasons were excluded. On the other hand, the inclusion criteria for end-users were limited to Bachelor of Medicine and Bachelor of Surgery (MBBS) students since they were the only undergraduate students available on campus during the semester break when the study was conducted. Postgraduate students, students enrolled in foundational studies, diplomas, executive diplomas, professional certificates, and short course programs were excluded from the study.

Recruitment

The recruitment of subject matter experts utilized the purposive sampling method. Only those who satisfied the specific criteria of having a minimum of 10 years of professional experience in the field of mental health were included. The experts received a formal invitation via email, which included an executive summary that provided information on the study's backgrounds, objectives, methodologies, and ethical considerations.

Likewise, MBBS students were recruited using the purposive sampling method. An official letter was emailed to the deputy deans of undergraduate and student affairs, seeking permission to conduct the research. The email included details about the research, similar to those distributed to experts. Upon receiving the approval of the faculty, the researcher contacted a professor from this professional network to schedule a 20-minute session for conducting a face validation study following the professor’s lecture to minimize any disruptions to her teaching.

Sample size calculations

Content validation studies recommend the inclusion of a panel consisting of two to 10 experts [20]. Hence, this study included the participation of five experts for script validation and nine experts for video validation. On the other hand, it is generally accepted that a minimum of 10 raters is adequate for face validity. However, the majority of studies included a standard sample size of 30 participants [21].

Study instruments

The instruments utilized in this study comprised sociodemographic data, the content validity index (CVI), and the face validity index (FVI). The sociodemographic data of the subject matter experts included age, gender, ethnicity, job title, and length of work experience. Conversely, the sociodemographic data of the end-users consisted of age, gender, nationality, marital status, ethnicity, enrolled programs, fields of study, and current academic semester and year.

The script and psychoeducational video were validated using the CVI and FVI. A panel of five experts evaluated every item of the script for its consistency, relevancy, representativeness, and clarity using a four-point Likert scale (1 = not relevant, 2 = somewhat relevant, 3 = quite relevant, and 4 = highly relevant). In contrast, a panel of nine experts evaluated the video's functionality, usability, efficiency, audiovisual style, and setting using a five-point Likert scale that consisted of responses ranging from 1 (strongly disagree), 2 (disagree), 3 (neutral), 4 (agree), and 5 (strongly agree). 

Before calculating the script’s CVI, items that received a rating of three or four were assigned a value of “1” (indicating validity), while those that received a rating of one or two were assigned a value of “0” (indicating invalidity). On the other hand, the CVI of the video was assigned a value of “1” (indicating validity) for items that received ratings of four or five and a value of “0” (indicating invalidity) for items that received ratings of one, two, or three.

The CVI was determined by calculating the item-level content validity index (I-CVI) and the scale-level item content validity index (S-CVI). The I-CVI was calculated by dividing the number of experts who rated the script as three or four and the video as four or five by the total number of experts. The values ranged from zero to one, with the script requiring an I-CVI of one to be validated following evaluation by a panel of three to five experts [20]. In contrast, a minimum I-CVI value of 0.78 was required for the video to be validated after an assessment by a panel of at least nine experts [20].

On the other hand, the S-CVI was calculated using the scale-level content validity index based on the average method (S-CVI/Ave) and the scale-level content validity index based on the universal agreement method (S-CVI/UA). The S-CVI/Ave was determined by summing the I-CVI values and dividing the sum by the total number of items [20]. On the other hand, the calculation of S-CVI/UA involved the summation of all items having an I-CVI value of 1, divided by the total number of items. Values >0.9 for S-CVI/Ave and >0.8 for S-CVI/UA were considered acceptable [20]. Nevertheless, the S-CVI/UA is affected by the number of experts, since a greater number of experts increases the probability of attaining a lower S-CVI value. This is due to the fact that the universal agreement (UA) score is assigned a value of 1 when the item achieves unanimous agreement from all experts. Other than that, the UA score is assigned a value of 0. Hence, the present study included I-CVI and S-CVI/Ave as per the recommendations suggested by Polit and Beck (2006) [22]. In addition, a designated space was allocated at the end of the questionnaire for experts to provide comments and recommendations.

The FVI was calculated using the same method as the CVI for video, with the exception that it was based on end-users’ perspectives. A group of 30 end-users evaluated the video's functionality, usability, efficiency, audiovisual style, and setting using a five-point Likert scale comparable to those used by experts, with responses ranging from 1 (strongly disagree), 2 (disagree), 3 (neutral), 4 (agree), and 5 (strongly agree). Prior to determining the FVI of the video, items with a rating of four or five were assigned the value "1" to signify validity, whereas items with a rating of one, two, or three were assigned the value "0" to indicate invalidity [23]. Similar to the content validity calculation, the FVI also includes the item-level face validity index (I-FVI), and the scale-level face validity index based on the average method (S-FVI/Ave) [23]. In order for the video to be considered validated, the FVI value has to meet a minimum threshold value of 0.80 [23]. Likewise, a designated area was made available at the end of the questionnaire for participants to provide any feedback or suggestions. The details of the instruments utilized in this research can be found in the appendices.

Data collection process

Script validation was conducted at the office of each expert. Prior to data collection, the researcher presented the experts with an overview of the study’s background, its objectives, methodology, and ethical conduct. A self-administered questionnaire was distributed, consisting of two sections. Section A comprised sociodemographic data, whereas Section B included the CVI questionnaire. Experts were provided with a two-week timeframe to complete the questionnaire, and the completed questionnaire was sent to the researcher via email. The validation of the psychoeducational video followed a similar approach, with the exception that experts completed the questionnaire immediately after watching the video. The estimated time to complete the questionnaire was approximately 15 minutes.

On the other hand, the face validation study was carried out among a sample of 30 fifth-year MBBS students in a classroom setting, using a method similar to CVI for video following their lectures. Prior to the commencement of the study, the researcher delivered a 10-minute PowerPoint presentation that provided an overview of the research's background, objectives, methodology, and ethical considerations. Subsequently, the students were presented with the psychoeducational video, and after it ended, they were handed a paper-based survey. It comprises two sections. Section A consisted of sociodemographic information, while Section B included the FVI questionnaire. The self-administered questions took approximately 15 minutes to complete. The completed forms were collected and securely stored in the researcher's repository.

Procedures

**Figure 1:**
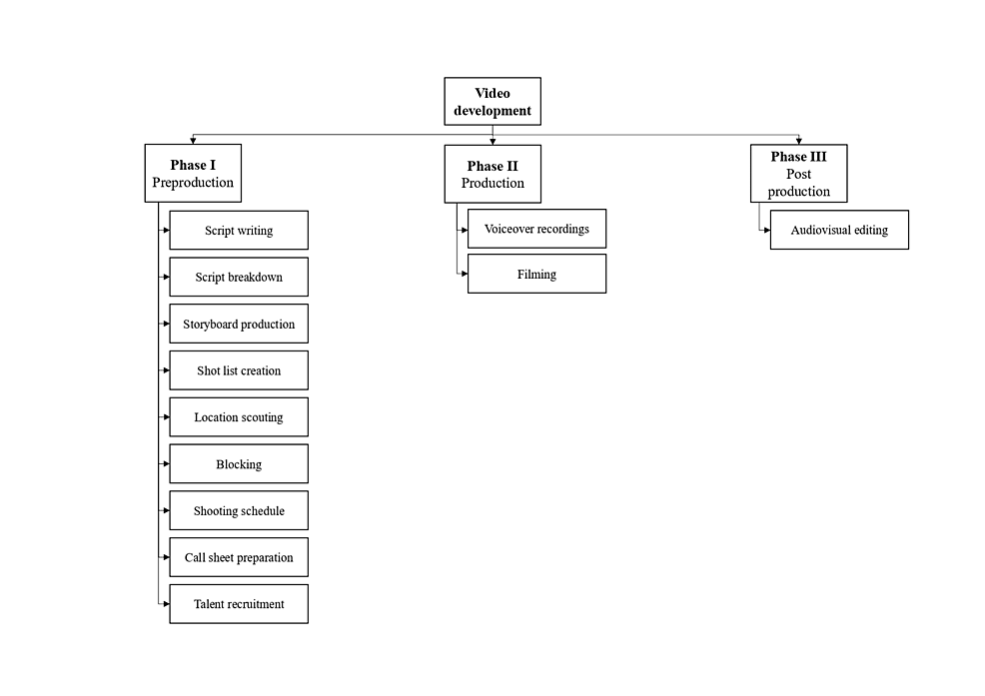
Video production processes

The psychoeducational video was developed following video production guidelines, which included preproduction, production, and postproduction phases, as shown in Figure 1 [24].

The preproduction phase consisted of scriptwriting, script breakdown, storyboard and shot list creation, location scouting, blocking, shooting schedule, call sheet preparation, and talent recruitment. The script was written in English using simple, easy-to-understand language to promote students’ engagement. The rationale for using English as the primary language of communication is to enable the researcher to reach a broader audience, which includes both domestic and international students. Furthermore, using English promotes uniformity and consistency in communication. The script’s content was based on medical reference books, such as the Fifth Edition of Key Concepts in Mental Health, Improving the Psychological Wellbeing of Children and Young People, the Fourth Edition of the Oxford Handbook of Psychiatry, and Desk Reference to the Diagnostic Criteria from DSM-5. Additionally, information from medically reviewed websites like Healthline and Mayo Clinic was also incorporated.

The topics covered 10 essential items outlined in the principles of psychoeducation. These items include the causes of depression, signs and symptoms of depression, recognizing early signs of relapse, coping mechanisms, treatment options, when and how to seek help, adhering to treatment, the long-term course and outcome of depression, guidelines for interacting with a depressed person, and dispelling myths, misconceptions, and stigma associated with depression [25]. Table 1 presents an outline of the video script's contents.

**Table 1:** The psychoeducational video’s content

No.	Items	Contents
1.	Causes of depression	Biological factors, genetic factors, psychological factors, environmental factors
2.	Common signs and symptoms of depression	Feeling worthless or intense guilt, lack of confidence and low self-esteem, sleeping too much or too little, overeating or a loss of interest in food, agitation, slowing of mental or physical processes, poor concentration
3.	Early recognition of depression relapse	A tendency to become more irritated and agitated, loss of all interest and activities, social isolation
4.	Coping strategies	Be open, spend time with family and friends, focus on positive aspects of life, do something you like
5.	Treatment for depression	Medications, psychotherapy, self-help, and coping
6.	When and how to seek help	When: unable to cope any longer, severe or ongoing symptoms that make it difficult to carry out daily activities; How: Division of Counseling and Disability Empowerment, Universiti Malaya, Talian HEAL 1555, Befrienders, MIASA Crisis Helpline
7.	The importance of adhering to treatment	Antidepressant withdrawal symptoms may result from sudden treatment cessation
8.	Long-term course and outcome of depression	Although depression is associated with poor outcomes, some patients do recover. Relapses of depression are common, even after many years of good health
9.	Dos and don’ts while dealing with a depressed person	Dos: encourage to speak up, assist them in finding help, offer to help with daily tasks; Don’ts: do not take it personally, avoid giving advice
10.	Clearing myths and misconceptions and dispelling stigma about depression	Depression and sadness are identical, depression does not affect man, and talking about depression only makes things worse

A group of five subject-matter experts consisting of two senior counselors, a public health medicine specialist, a psychiatrist, and an educational psychology and counseling professor validated the script to ensure its consistency, relevancy, representativeness, and clarity. Subsequently, a bilingual language expert translated the validated script into Malay to create subtitles, with the aim of increasing understanding for native speakers who may not be fluent in English. A script breakdown was conducted to determine the necessary components needed for production, such as the number of actors, types of props, and filming locations. Subsequently, the researcher and the university's video production team, consisting of experts in broadcasting, content development, graphic design, and video editing, worked together to produce the storyboard. The storyboard was produced using a PowerPoint storyboard template. It includes shot type, dialogue, direction arrows to illustrate character movement, camera movements, and other descriptive information to outline the sequential progression of the video. Subsequently, the shot list was produced using the script and storyboards for capturing the necessary footage, helping the editor arrange the shots during postproduction, and allowing the director to estimate the filming time.

Suitable filming locations were selected, comprising both indoor and outdoor settings. This included the university's outdoor spaces, classrooms, and counseling department, with the aim of enhancing its visual attractiveness. Furthermore, the technique of coordinating actors' movements in relation to the camera, known as blocking, was selected. In keeping with the overall concept of the video, the actor remained still, and the camera remained fixed to block the scene. The researcher also received detailed information from the director regarding the shooting schedule, which includes the filming timetable and location. The final component in the preproduction stage was talent recruitment. The researcher recruited a total of seven talents from his network of contacts. This group of actors was comprised of individuals who voluntarily participated and were selected based on their proficiency in English and suitability for the roles of hosts, depressed students, counselors, and supporting actors. They consisted of university students, medical faculty, and counseling division staff members.

The video production spanned a period of three days, as planned in advance during the preproduction phase. It involved voiceover recordings and filming that was carried out with the assistance of the university’s video production staff. The hosts’ voiceover recordings were conducted at the university's recording studio using Adobe Audition software at an optimal speed for audiobook narration, ranging from 150 to 160 words per minute. The filming was carried out using a Sony A7s2 camera paired with a 24-70 mm G lens. A Heroview 24" teleprompter was used for script viewing, while a Sony Clip Mic was used for sound recording. The video was recorded following the rule of thirds in 1080p resolution with a frame rate of 50 frames per second to enhance balance and provide a seamless and sharp image. Lighting and shadow adjustments were applied to add depth and richness, resulting in a visually compelling experience. The headroom and noseroom, camera angles, and shot types, including long-shot, medium-shot, and close-up, were adjusted to optimize the video’s outcome.

During the postproduction phase, the video was enhanced by using cuts and dissolve transition effects, along with licensed background music, to evoke emotion and improve the overall presentation. The visual editing was carried out using the Recorder Footage and Lumafusion software, while the stock videos were acquired from licensed Envato Elements and edited using Adobe Premiere 2022. Additionally, the Adobe After Effects 2023 software was used to create the pre-finalized version of the psychoeducational video by applying motion design and image tilting techniques. Nine experts and 30 end-users evaluated the pre-finalized version of the psychoeducational video to determine its functionality, usability, efficiency, audiovisual style, and settings. The experts consisted of the participants from the script's content validation study, as well as four additional subject matter experts comprising two primary care medicine specialists, a public health medicine specialist, and a psychiatrist.

## Results

Demographic profile of the participants

The mean age of the subject matter experts was 45.78 years, and their mean work experience was 20.75 years. 22% of the experts were male, while 78% were female. Conversely, the mean age of the end-users was 24.26 years. 60% of the participants were female, while 40% were male.

Content validity index

The CVI findings for the script indicated a score of one for I-CVI and S-CVI/Ave in terms of consistency, relevancy, representativeness, and clarity, as shown in Table 2.

**Table 2:** Evaluation of the video script (consistency, relevancy, representativeness, and clarity domains) by five experts Items rated as three or four were recoded as valid (1); items rated as one or two were recoded as non-valid (0); acceptable CVI values = 1. I-CVI, item-level content validity index; S-CVI/Ave, scale-level content validity index based on the average method

Items	Rater 1	Rater 2	Rater 3	Rater 4	Rater 5	Experts in agreement	I-CVI
Consistency							
1	1	1	1	1	1	5	1
2	1	1	1	1	1	5	1
3	1	1	1	1	1	5	1
4	1	1	1	1	1	5	1
5	1	1	1	1	1	5	1
6	1	1	1	1	1	5	1
7	1	1	1	1	1	5	1
8	1	1	1	1	1	5	1
9	1	1	1	1	1	5	1
10	1	1	1	1	1	5	1
Proportion	1	1	1	1	1	S-CVI/Ave	1
Relevancy							
1	1	1	1	1	1	5	1
2	1	1	1	1	1	5	1
3	1	1	1	1	1	5	1
4	1	1	1	1	1	5	1
5	1	1	1	1	1	5	1
6	1	1	1	1	1	5	1
7	1	1	1	1	1	5	1
8	1	1	1	1	1	5	1
9	1	1	1	1	1	5	1
10	1	1	1	1	1	5	1
Proportion	1	1	1	1	1	S-CVI/Ave	1
Representativeness							
1	1	1	1	1	1	5	1
2	1	1	1	1	1	5	1
3	1	1	1	1	1	5	1
4	1	1	1	1	1	5	1
5	1	1	1	1	1	5	1
6	1	1	1	1	1	5	1
7	1	1	1	1	1	5	1
8	1	1	1	1	1	5	1
9	1	1	1	1	1	5	1
10	1	1	1	1	1	5	1
Proportion	1	1	1	1	1	S-CVI/Ave	1
Clarity							
1	1	1	1	1	1	5	1
2	1	1	1	1	1	5	1
3	1	1	1	1	1	5	1
4	1	1	1	1	1	5	1
5	1	1	1	1	1	5	1
6	1	1	1	1	1	5	1
7	1	1	1	1	1	5	1
8	1	1	1	1	1	5	1
9	1	1	1	1	1	5	1
10	1	1	1	1	1	5	1
Proportion	1	1	1	1	1	S-CVI/Ave	1

In contrast, the I-CVI for the psychoeducational video ranged between 0.78 and 1, which satisfies the minimum acceptable value of 0.78. Similarly, the S-CVI/Ave for the domains of functionality, usability, efficacy, audiovisual technique, and setting varies between 0.92 and 0.98, which also meets the minimum acceptable value of 0.9. These values are presented in Table 3.

**Table 3:** Evaluation of the psychoeducational video (functionality, usability, efficacy, audiovisual technique, and setting domains) by nine experts Items rated as four or five were recoded as valid (1); items rated as one, two, or three were recoded as non-valid (0); acceptable I-CVI value (> 0.78) and S-CVI/Ave value (>0.9). I-CVI, item-level content validity index; S-CVI/Ave: scale-level content validity index based on the average method

Items	Rater 1	Rater 2	Rater 3	Rater 4	Rater 5	Rater 6	Rater 7	Rater 8	Rater 9	Experts in agreement	I-CVI
Functionality											
1	1	1	1	1	1	1	1	1	1	9	1
2	1	1	1	1	1	1	1	1	1	9	1
3	1	1	1	1	1	1	1	1	1	9	1
4	1	1	1	1	0	1	1	1	1	8	0.89
5	1	1	1	1	1	1	1	1	1	9	1
6	1	1	1	1	1	1	1	1	1	9	1
7	1	1	1	1	1	1	1	1	1	9	1
8	1	1	1	1	1	0	1	1	1	8	0.89
9	1	1	1	1	1	1	1	1	1	9	1
10	1	1	1	1	1	1	1	1	1	9	1
Proportion	1	1	1	1	0.9	0.9	1	1	1	S-CVI/Ave	0.98
Usability											
1	1	1	0	1	1	1	1	1	1	8	0.89
2	1	1	1	1	1	1	1	1	1	9	1
3	1	1	1	1	1	1	1	1	1	9	1
4	1	1	1	1	0	1	1	1	1	8	0.89
5	0	1	1	1	1	1	1	1	1	8	0.89
6	1	1	1	1	1	1	1	1	1	9	1
7	0	1	1	1	1	1	1	1	1	8	0.89
8	1	1	1	1	1	0	1	1	1	8	0.89
9	1	1	1	1	1	1	1	1	1	9	1
10	1	1	1	1	1	1	1	1	1	9	1
Proportion	0.8	1	0.9	1	0.9	0.9	1	1	1	S-CVI/Ave	0.95
Efficiency											
1	1	1	1	0	1	1	1	1	1	8	0.89
2	1	1	1	1	1	1	1	1	1	9	1
3	1	1	1	1	1	1	1	1	1	9	1
4	1	1	1	1	0	1	1	1	1	8	0.89
5	0	1	1	1	1	0	1	1	1	7	0.78
6	1	1	1	1	1	1	1	1	1	9	1
7	0	1	1	1	1	0	1	1	1	7	0.78
8	1	1	1	1	1	0	1	1	1	8	0.89
9	1	1	1	1	1	1	1	1	1	9	1
10	1	1	1	1	1	1	1	1	1	9	1
Proportion	0.8	1	1	0.9	0.9	0.7	1	1	1	S-CVI/Ave	0.92
Audiovisual technique											
1	1	1	1	1	1	1	1	1	1	9	1
2	1	1	1	1	1	1	1	1	1	9	1
3	1	1	1	1	1	1	1	1	1	9	1
4	1	1	1	1	0	1	1	1	1	8	0.89
5	1	1	1	1	1	1	1	1	1	9	1
6	1	1	1	1	1	1	1	1	1	9	1
7	1	1	1	1	1	1	1	1	1	9	1
8	1	1	1	1	1	0	1	1	1	8	0.89
9	1	1	1	1	1	1	1	1	1	9	1
10	1	1	1	1	1	1	1	1	1	9	1
Proportion	1	1	1	1	0.9	0.9	1	1	1	S-CVI/Ave	0.98
Settings											
1	1	1	1	0	1	1	1	1	0	7	0.78
2	1	1	1	1	1	1	1	1	1	9	1
3	1	1	1	1	1	1	1	1	1	9	1
4	1	1	1	1	0	1	1	1	1	8	0.89
5	1	1	1	1	1	1	1	1	1	9	1
6	1	1	1	1	1	1	1	1	1	9	1
7	1	1	1	1	1	1	1	1	1	9	1
8	1	1	1	1	1	0	1	1	1	8	0.89
9	1	1	1	0	1	1	1	1	1	8	0.89
10	1	1	1	1	1	1	1	1	1	9	1
Proportion	1	1	1	0.8	0.9	0.9	1	1	0.9	S-CVI/Ave	0.94

Face validity index

The findings indicated that the I-FVI exceeded the minimum acceptable threshold of 0.80 (with a range of 0.87 to 1) in the categories of functionality, usability, efficacy, audiovisual technique, and setting. Likewise, the S-FVI/Ave values for all categories exceeded the acceptable cut-off point of 0.80. The S-FVI/Ave values for functionality, usability, and efficacy were 0.91, 0.96, and 0.97, respectively, while those for audiovisual technique and setting were 0.94 and 0.98. The results are shown in Table 4.

**Table 4:** Evaluation of the psychoeducational video (functionality, usability, efficacy, audiovisual technique, and setting domains) by 30 end-users Items rated as four or five were recoded as valid (1); items rated as one, two, or three were recoded as non-valid (0); acceptable FVI values >0.80. I, items; R, rater; RA, raters in agreement; P, proportion; I-FVI, item-level face validity index; S-FVI/Ave, scale-level face validity index based on the average method

I	R 1	R 2	R 3	R 4	R 5	R 6	R 7	R 8	R 9	R 10	R 11	R 12	R 13	R 14	R 15	R 16	R 17	R 18	R 19	R 20	R 21	R 22	R 23	R 24	R 25	R 26	R 27	R 28	R 29	R 30	RA	I-FVI
Functionality																																
1	1	1	1	1	1	1	1	1	1	1	1	1	1	1	1	1	1	1	1	1	1	1	1	1	1	1	1	1	1	1	30	1
2	0	1	1	1	1	1	1	1	1	1	1	1	1	1	1	0	1	1	1	1	1	1	1	1	1	0	1	1	1	1	27	0.9
3	1	1	1	1	1	1	1	1	1	1	1	1	1	1	1	1	1	1	1	1	1	1	1	1	1	0	1	1	1	1	29	0.97
4	1	1	1	1	1	1	1	1	1	1	1	1	1	1	1	1	1	1	1	1	1	1	1	1	0	1	1	1	1	1	29	0.97
5	1	1	1	1	1	1	1	1	1	1	1	1	1	1	0	1	1	1	1	1	1	1	1	1	1	1	1	1	1	1	29	0.97
6	1	1	1	1	1	1	1	1	1	1	1	1	1	1	1	1	1	1	1	1	1	1	1	1	1	1	1	1	1	1	30	1
7	1	1	1	1	1	1	1	1	1	1	1	1	1	1	0	1	1	1	1	1	1	1	1	1	1	1	1	1	1	1	29	0.97
8	1	1	1	1	1	0	1	1	1	1	1	1	1	1	1	1	1	1	1	1	1	1	1	1	1	1	1	1	1	1	29	0.97
9	1	1	1	1	1	0	1	1	1	1	1	1	1	1	0	0	1	1	1	1	1	1	1	1	1	1	1	1	1	1	27	0.9
10	1	1	1	1	1	1	1	1	1	1	1	1	1	1	1	1	1	1	1	1	1	1	1	1	0	0	1	1	1	1	28	0.93
P	0.9	1	1	1	1	0.8	1	1	1	1	1	1	1	1	0.7	0.8	1	1	1	1	1	1	1	1	0.8	0.7	1	1	1	1	S-FVI/Ave	0.91
Usability																																
1	0	1	1	1	1	1	1	1	1	1	1	1	1	1	1	1	1	1	1	1	1	1	1	1	1	0	1	1	1	1	28	0.93
2	1	1	1	1	1	1	1	1	1	1	1	1	1	1	1	0	1	1	1	1	1	1	1	1	1	1	1	1	1	1	29	0.97
3	1	1	1	1	1	1	1	1	1	1	1	1	1	1	1	1	1	1	1	1	1	1	1	1	1	1	1	1	1	1	30	1
4	1	1	1	1	1	1	1	1	1	1	1	1	1	1	1	1	1	1	1	1	1	1	1	1	1	1	1	1	1	1	30	1
5	1	1	1	1	1	1	1	1	1	1	1	1	1	1	0	1	1	1	1	1	1	1	1	0	1	1	1	1	1	1	28	0.93
6	1	1	1	1	1	1	1	1	1	1	1	1	1	1	1	1	1	1	1	1	1	1	1	1	1	1	1	1	1	1	30	1
7	1	1	1	1	1	1	1	1	1	1	1	1	1	1	0	1	1	1	1	1	1	1	1	0	1	1	1	1	1	1	28	0.93
8	1	1	1	1	1	1	1	1	1	1	1	1	1	1	1	1	1	1	1	1	1	1	1	1	1	1	1	1	1	1	30	1
9	1	1	1	1	1	1	1	1	1	1	1	1	1	1	0	0	1	1	1	1	1	1	1	1	0	1	1	1	1	1	27	0.9
10	1	1	1	1	1	1	1	1	1	1	1	1	1	1	1	1	1	1	1	1	1	1	1	1	0	0	1	1	1	1	28	0.93
P	0.9	1	1	1	1	1	1	1	1	1	1	1	1	1	0.7	0.8	1	1	1	1	1	1	1	0.8	0.8	0.8	1	1	1	1	S-FVI/Ave	0.96
Efficiency																																
1	1	1	1	1	1	1	1	1	1	1	1	1	1	1	1	1	1	1	1	1	1	1	1	1	1	1	1	1	1	1	30	1
2	1	1	1	1	1	1	1	1	1	1	1	1	1	1	1	1	1	1	1	1	1	1	1	1	1	1	1	1	1	1	30	1
3	1	1	1	1	1	1	1	1	1	1	1	1	1	1	1	1	1	1	1	1	1	1	1	1	1	1	1	1	1	1	30	1
4	1	1	1	1	1	1	1	1	1	0	1	1	1	1	1	1	1	1	1	1	1	1	1	1	0	1	1	1	1	1	28	0.93
5	1	1	1	1	1	1	1	1	1	1	1	1	1	1	0	1	1	1	1	1	1	1	1	1	0	1	1	1	1	1	28	0.93
6	1	1	1	1	1	1	1	1	1	1	1	1	1	1	1	1	1	1	1	1	1	1	1	1	1	1	1	1	1	1	30	1
7	1	1	1	1	1	1	1	1	1	1	1	1	1	1	0	1	1	1	1	1	1	1	1	1	0	1	1	1	1	1	28	0.93
8	1	1	1	1	1	1	1	1	1	1	1	1	1	1	1	1	1	1	1	1	1	1	1	1	1	1	1	1	1	1	30	1
9	1	1	1	1	1	1	1	1	1	0	1	1	1	1	0	0	1	1	1	1	1	1	1	1	0	1	1	1	1	1	26	0.87
10	1	1	1	1	1	1	1	1	1	1	1	1	1	1	1	1	1	1	1	1	1	1	1	1	1	1	1	1	1	1	30	1
P	1	1	1	1	1	1	1	1	1	0.8	1	1	1	1	0.7	0.9	1	1	1	1	1	1	1	1	0.6	1	1	1	1	1	S-FVI/Ave	0.97
Audiovisual technique																																
1	0	1	1	1	1	1	1	1	1	1	0	1	1	1	1	1	1	1	1	1	1	1	1	1	1	1	1	1	0	1	27	0.9
2	1	1	1	1	1	1	1	1	1	1	0	1	1	1	1	1	1	1	1	1	1	1	1	1	1	1	1	1	1	1	29	0.97
3	1	1	1	1	1	1	1	1	1	1	0	1	1	1	1	1	1	1	1	1	1	1	1	1	1	1	1	1	1	1	29	0.97
4	1	1	1	1	1	1	1	1	1	1	1	1	1	1	1	1	1	1	1	1	1	1	1	1	1	1	1	1	0	1	29	0.97
5	1	1	1	1	1	1	1	1	1	0	1	1	1	1	0	1	1	1	1	1	1	1	1	1	1	1	1	0	1	1	27	0.9
6	1	1	1	1	1	1	1	1	1	1	1	1	1	1	1	1	1	1	1	1	1	1	1	1	1	1	1	1	1	1	30	1
7	1	1	1	1	1	1	1	1	1	0	1	1	1	1	0	1	1	1	1	1	1	1	1	1	1	1	1	0	1	1	27	0.9
8	1	1	1	1	1	1	1	1	1	1	0	1	1	1	1	1	1	1	1	1	1	1	1	1	1	1	1	1	1	1	29	0.97
9	1	1	1	1	1	1	1	1	1	1	1	1	1	1	0	0	1	1	1	1	1	1	1	1	0	1	0	1	1	1	26	0.87
10	1	1	1	1	1	1	1	1	1	1	0	1	1	1	1	1	1	1	1	1	1	1	1	1	1	1	1	1	1	1	29	0.97
P	0.9	1	1	1	1	1	1	1	1	0.8	0.5	1	1	1	0.7	0.9	1	1	1	1	1	1	1	1	0.9	1	0.9	0.8	0.8	1	S-FVI/Ave	0.94
Settings																																
1	1	1	1	1	1	1	1	1	1	1	1	1	1	1	1	1	1	1	1	1	1	1	1	1	1	1	1	1	1	1	30	1
2	1	1	1	1	1	1	1	1	1	1	1	1	1	1	1	1	1	1	1	1	1	1	1	1	1	1	1	1	1	1	30	1
3	1	1	1	1	1	1	1	1	1	1	1	1	1	1	1	1	1	1	1	1	1	1	1	1	1	1	1	1	1	1	30	1
4	1	1	1	1	1	1	1	1	1	1	1	1	1	1	1	1	1	1	1	1	1	1	1	1	1	1	1	1	1	1	30	1
5	1	1	1	1	1	1	1	1	1	1	1	1	1	1	0	1	1	1	1	1	1	1	1	1	1	1	1	1	1	1	29	0.97
6	1	1	1	1	1	1	1	1	1	1	1	1	1	1	1	1	1	1	1	1	1	1	1	1	1	1	1	1	1	1	30	1
7	1	1	1	1	1	1	1	1	1	1	1	1	1	1	0	1	1	1	1	1	1	1	1	1	1	1	1	1	1	1	29	0.97
8	1	1	1	1	1	1	1	1	1	1	1	1	1	1	1	1	1	1	1	1	1	1	1	1	1	1	1	1	1	1	30	1
9	1	1	1	1	1	1	1	1	1	1	1	1	1	1	0	0	1	1	1	1	1	1	1	1	0	1	1	1	1	1	27	0.9
10	1	1	1	1	1	1	1	1	1	1	1	1	1	1	1	1	1	1	1	1	1	1	1	1	1	1	1	1	1	1	30	1
P	1	1	1	1	1	1	1	1	1	1	1	1	1	1	0.7	0.9	1	1	1	1	1	1	1	1	0.9	1	1	1	1	1	S-FVI/Ave	0.98

Participants’ feedback

Feedback from participants indicated that the psychoeducational video was captivating and had the potential to improve depression literacy among university students. However, several recommendations were proposed to improve the video. The first recommendation was to reduce the speed of the video to increase the readability of the subtitles, as it was believed that the video’s speed could make it difficult for viewers to read them. Second, it was recommended to replace the gloomy tone of the background music with a vibrant composition and tempo to create a more pleasant and engaging mood. Finally, it was recommended to replace stock videos featuring Caucasian actors in Western settings with footage that portrays Asian settings.

The feedback was carefully evaluated by both the researcher and the video production team prior to its incorporation into the video. However, the participants' recommendation to reduce the video's speed with the aim of improving the readability of the subtitles was not implemented following a discussion with the video director and the rest of the video production team. The rationale for this decision was that prolonging the video's runtime would lead to a reduction in the audience's attention span and a loss of engagement with the video's content. The finalized video had a duration of 11 minutes and 19 seconds. It was subsequently encoded using Adobe Media Encoder 2022 and posted to the university’s official YouTube account channel, which serves as the main platform for video distribution. The video can be accessed using the following link: https://www.youtube.com/watch?v=8cO2-GohPJk.

## Discussion

This study highlighted the systematic approach to video development, including the validation of the script and video by subject matter experts and end-users. The high CVI and FVI scores provide strong evidence that the psychoeducational video is a valid tool for improving depression literacy among university students. Recognizing the importance of developing and validating educational materials is crucial for establishing a scientifically reliable tool. This is because educational materials that have been meticulously developed and validated have the potential to have a major impact on their target audiences [26]. For example, a randomized controlled trial study using a psychoeducational video titled "Depression in Portuguese University Students" found that the video was effective in improving university students' knowledge of depression [27]. This was based on the results of the Scheffé and Least Significant Difference multiple comparisons tests, which revealed that university students who received the audiovisual intervention had significantly higher post-intervention depression knowledge scores than those who received the narrative text or news format [27].

Participants recommended reducing the speed of the video in order to improve the readability of the subtitles. However, this would extend the video’s duration, which could reduce students’ attention and ability to retain information, as well as increase the risk of mental distractions [28]. Additionally, the video's speed adhered to the "six-second rule," in which a two-line subtitle was displayed on screen for six seconds, allowing the audience sufficient time to read it. Studies have shown that viewers are able to understand fast-paced subtitles without losing any focus on the visual components [29]. Moreover, the use of slower subtitles does not improve knowledge retention and may result in the need to read the subtitles a second time, thereby reducing the level of engagement and enjoyment throughout the viewing experience [29].

Background music in an educational video is important as it significantly impacts the cognitive processes of viewers, especially those associated with visual perception. Participants suggested substituting the gloomy background music with a more energetic and vibrant musical arrangement. Nevertheless, the tempo of background music has no significant impact on capturing the audience's attention [30]. This finding was based on a brainwave indicator that measured attentional processes, which revealed no significant changes in attention levels among participants when they were exposed to various tempos of background music [30].

The video was intended to provide a detailed representation of Malaysian university settings. As a result, the production employed actors who were university students and staff members from within the institution, filmed on the university campus, and incorporated stock video footage that portrayed Asian culture. Research has suggested that integrating a local context into educational videos can help promote student engagement by emphasizing that the video is specifically designed for the students. Additionally, it has the potential to be used in future semesters [28].

It is important to note that the study was conducted in two phases: development and validation, similar to Rosa B et al.’s (2019) research, which aimed to develop and validate an educational video on colostomy caused by cancer while adhering to all recommended methodological steps for its production [26]. This approach ensured compliance with established standards for producing a high-quality video and provided a comprehensive set of instructions and best practices that could benefit future researchers in creating video-based interventions. Furthermore, the video's content, based on the principles of psychoeducation, was compiled from evidence-based materials and validated by experienced subject-matter experts, ensuring that viewers receive accurate and reliable information. 

Nevertheless, this research encountered several limitations. The sample selected for the face validation study is limited in terms of generalizability, as it only includes final-year MBBS students. Hence, it is crucial to exercise caution when interpreting the findings, as final-year MBBS students have a higher level of depression literacy in comparison to undergraduates from other faculties. Therefore, their opinions may differ from those of other undergraduates who have limited or no knowledge of depression. Thus, it is recommended that future studies on face validation include participants from various faculties.

In addition, the participation of screen production experts in this study was limited as a result of their work commitments. Nevertheless, the subject matter experts were capable of evaluating both the content and its audiovisual components based on their previous experience overseeing video-based interventions. Therefore, it is recommended for future studies to include screen production experts who may provide valuable insights into the development and validation of the video’s audiovisual components. This has the potential to improve the video’s credibility and reliability as an educational tool. Moreover, the poor participation from students and faculty members due to their shyness, nervousness, self-consciousness, and lack of self-confidence to act in front of the camera has limited the amount of footage that can be captured. As a result, it had to be substituted with stock video footage.

## Conclusions

The psychoeducational video entitled "Educating University Students About Depression: A Short Documentary" was systematically developed in accordance with the principles of psychoeducation and the established guidelines for video production. The video included evidence-based content, feedback from participants, and film settings tailored to the target audience. Additionally, the video was determined to be valid following an evaluation by subject-matter experts and end-users. Therefore, it has the ability to educate and improve the literacy of university students regarding depression.

Improvements in depression literacy have the potential to mitigate the adverse consequences of depression among university students, such as suicidal ideation, substance abuse, and other related issues. Moreover, it may have the ability to help students recognize the signs and symptoms of depression in their peers and guide them to seek professional help. This would create a strong sense of community support among the students.

## Appendices

Script content validation study

Title: Educating University Students About Depression: A Short Documentary

Dear expert,

This survey is divided into two sections: sociodemographic data and a content validation form. We would appreciate your expert input on the **consistency, relevancy, representativeness,** and **clarity **of each script element. It is important to note that your review should be based on the concept of psychoeducation pertaining to depression. We kindly request that you adopt a neutral and constructive approach in your review and use the following rating scale:

1 = Not relevant

2 = Somewhat relevant

3 = Quite relevant

4 = Highly relevant

Any additional feedback or suggestions are greatly appreciated.

The information will be kept strictly confidential

Section A: Experts' Sociodemographic Data

Please answer all the following questions. Use (✓ ) to mark your answer

**Table 5:** Experts' sociodemographic data for script content validity study

No.	Question	Official use
1	Age: ___ years	
2	Gender: Male Female	
3	Ethnicity: Malay Chinese Indian Others. If others, please specify ___	
4	What is your current job position?: Public health specialist Psychologist Counsellor Others. If others, please specify ___	
5	How long have you been employed in this field? Please state: ___ years	
6	What is the duration of your experience in managing mental illness associated with depression? Please state: ___ years	

Section B: Content Validation Form (Script)

**Table 6:** Form for script content validation study

Items	Is the information consistent across each component of the script?				Is the script’s content relevant to the concept of psychoeducation for depression?				Does each component of the script represent the concept of psychoeducation for depression?				Is the script’s wording clear?			
Etiological factors	1	2	3	4	1	2	3	4	1	2	3	4	1	2	3	4
Common signs and symptoms	1	2	3	4	1	2	3	4	1	2	3	4	1	2	3	4
Awareness regarding the early sign of relapse	1	2	3	4	1	2	3	4	1	2	3	4	1	2	3	4
Hope to cope with the situation	1	2	3	4	1	2	3	4	1	2	3	4	1	2	3	4
Treatment	1	2	3	4	1	2	3	4	1	2	3	4	1	2	3	4
When and how to seek treatment	1	2	3	4	1	2	3	4	1	2	3	4	1	2	3	4
The importance of adhering to treatment	1	2	3	4	1	2	3	4	1	2	3	4	1	2	3	4
Long-term course and outcome	1	2	3	4	1	2	3	4	1	2	3	4	1	2	3	4
Dos and don’ts while dealing with the patient	1	2	3	4	1	2	3	4	1	2	3	4	1	2	3	4
Clearing myths and misconceptions about the illness and dispelling stigma	1	2	3	4	1	2	3	4	1	2	3	4	1	2	3	4

Comments: _______________________________________________________________________________________________________________________________________________________________________________________________________________________________________________________________________________________________________________________________________________________________________________________

Video content validation study

Title: Educating University Students About Depression: A Short Documentary

Dear expert,

This survey is divided into two sections: sociodemographic data and a content validation form. We would appreciate your expert input on the **functionality, usability, efficiency, audiovisual technique,** and **setting **of each video element. It is important to note that your review should based on the concept of psychoeducation pertaining to depression. We kindly request that you adopt a neutral and constructive approach in your review and use the following rating scale:

1 = Totally disagree 

2 = Disagree

3 = Neither agree nor disagree 

4 = Agree

5 = Totally agree

Any additional feedback or suggestions are greatly appreciated.

The information will be kept strictly confidential.

Section A: Experts’ Sociodemographic Data

Please answer all the following questions. Use (✓ ) to mark your answer

**Table 7:** Experts' sociodemographic data for video content validity study

No.	Question	Official use
	Age: _____ years	
	Gender: Male Female	
	Ethnicity: Malay Chinese Indian Others If others, please specify_________________________________	
	What is your job position?: Public Health Specialist Psychologist Counsellor Others If others, please specify_________________________________	
	For how long have you been working in this profession?: Please state: ______ years	
	Experience managing depression-related mental illness or public health issues associated with mental health. Please state: ______ years	

Section B: Content Validation Form (Video)

**Table 8:** Form for video content validation study

Items	Does each item's content serve its intended purpose?					Does the video's content help to increase literacy in relation to each content?					Does the video effectively improve people’s understanding of each of its contents?					Is the video's audiovisual technique suitable for each of its contents?					Are the settings of the video appropriate for presenting each of its contents?				
Etiological factors	1	2	3	4	5	1	2	3	4	5	1	2	3	4	5	1	2	3	4	5	1	2	3	4	5
Common signs and symptoms	1	2	3	4	5	1	2	3	4	5	1	2	3	4	5	1	2	3	4	5	1	2	3	4	5
Awareness regarding the early sign of relapse	1	2	3	4	5	1	2	3	4	5	1	2	3	4	5	1	2	3	4	5	1	2	3	4	5
Hope to cope with the situation	1	2	3	4	5	1	2	3	4	5	1	2	3	4	5	1	2	3	4	5	1	2	3	4	5
Treatment	1	2	3	4	5	1	2	3	4	5	1	2	3	4	5	1	2	3	4	5	1	2	3	4	5
When and how to seek treatment	1	2	3	4	5	1	2	3	4	5	1	2	3	4	5	1	2	3	4	5	1	2	3	4	5
The importance of adhering to treatment	1	2	3	4	5	1	2	3	4	5	1	2	3	4	5	1	2	3	4	5	1	2	3	4	5
Long-term course and outcome	1	2	3	4	5	1	2	3	4	5	1	2	3	4	5	1	2	3	4	5	1	2	3	4	5
Dos and don’ts while dealing with the patient	1	2	3	4	5	1	2	3	4	5	1	2	3	4	5	1	2	3	4	5	1	2	3	4	5
Clearing myths and misconceptions about the illness and dispelling stigma	1	2	3	4	5	1	2	3	4	5	1	2	3	4	5	1	2	3	4	5	1	2	3	4	5

Comments: _______________________________________________________________________________________________________________________________________________________________________________________________________________________________________________________________________________________________________________________________________________________________________________________

Video face validation study

Title: Educating University Students About Depression: A Short Documentary

Dear students,

This survey is divided into two sections: sociodemographic data and a content validation form. We would appreciate your expert input on the **functionality, usability, efficiency, audiovisual technique,** and **setting **of each video element. It is important to note that your review should be based on the concept of the video's topic. We kindly request that you adopt a neutral and constructive approach in your review and use the following rating scale:

1 = Totally disagree 

2 = Disagree

3 = Neither agree nor disagree 

4 = Agree

5 = Totally agree

Any additional feedback or suggestions are greatly appreciated.

The information will be kept strictly confidential.

Section A: End-Users’ Sociodemographic Data

Please answer all the following questions. Use (✓ ) to mark your answer

**Table 9:** End-users' sociodemographic data for video face validity study

No.	Question	Official use
1	Age: ___ years	
2	Gender: Male Female	
3	Nationalities: Citizen Non-citizen	
4	Marital status: Never married Married Widowed Divorced Separated	
5	Ethnicity: Malay Chinese Indian Others. If others, please specify ___	
6	Enrolled programmes: ___	
7	Field of study: ___	
8	Current academic semester:___	
9	Current academic year: ___	

Section B: Face Validation Form (Video)

**Table 10:** Form for video face validation study

Items	Does each item's content serve its intended purpose?					Does the video's content help to increase literacy in relation to each content?					Does the video effectively improve people’s understanding of each of its contents?					Is the video's audiovisual technique suitable for each of its contents?					Are the settings of the video appropriate for presenting each of its contents?				
Etiological factors	1	2	3	4	5	1	2	3	4	5	1	2	3	4	5	1	2	3	4	5	1	2	3	4	5
Common signs and symptoms	1	2	3	4	5	1	2	3	4	5	1	2	3	4	5	1	2	3	4	5	1	2	3	4	5
Awareness regarding the early sign of relapse	1	2	3	4	5	1	2	3	4	5	1	2	3	4	5	1	2	3	4	5	1	2	3	4	5
Hope to cope with the situation	1	2	3	4	5	1	2	3	4	5	1	2	3	4	5	1	2	3	4	5	1	2	3	4	5
Treatment	1	2	3	4	5	1	2	3	4	5	1	2	3	4	5	1	2	3	4	5	1	2	3	4	5
When and how to seek treatment	1	2	3	4	5	1	2	3	4	5	1	2	3	4	5	1	2	3	4	5	1	2	3	4	5
The importance of adhering to treatment	1	2	3	4	5	1	2	3	4	5	1	2	3	4	5	1	2	3	4	5	1	2	3	4	5
Long-term course and outcome	1	2	3	4	5	1	2	3	4	5	1	2	3	4	5	1	2	3	4	5	1	2	3	4	5
Dos and don’ts while dealing with the patient	1	2	3	4	5	1	2	3	4	5	1	2	3	4	5	1	2	3	4	5	1	2	3	4	5
Clearing myths and misconceptions about the illness and dispelling stigma	1	2	3	4	5	1	2	3	4	5	1	2	3	4	5	1	2	3	4	5	1	2	3	4	5FV

Comments: _______________________________________________________________________________________________________________________________________________________________________________________________________________________________________________________________________________________________________________________________________________________________________________________
